# The Effects of Prenatal Diagnosis on the Interaction of the Mother–Infant Dyad: A Longitudinal Study of Prenatal Care in the First Year of Life

**DOI:** 10.3389/fpsyg.2022.804724

**Published:** 2022-03-28

**Authors:** Vera Cristina Alexandre de Souza, Erika Parlato-Oliveira, Lêni Márcia Anchieta, Alexei Manso Correa Machado, Sylvie Viaux Savelon

**Affiliations:** ^1^School of Medicine, Universidade Federal de Minas Gerais, Belo Horizonte, Brazil; ^2^Centre de Recherche Psychanalyse, Médecine et Société (CRPMS), Université de Paris, Paris, France; ^3^École Doctorale – UFR d’Études Psychanalytiques, Université de Paris, Paris, France; ^4^Department of Anatomy and Image, School of Medicine, Federal University of Minas Gerais, Belo Horizonte, Brazil; ^5^Graduate Program in Informatics, Pontifical Catholic University of Minas Gerais, Belo Horizonte, Brazil; ^6^Neonatal and Obstetrical Department, University Hospital Croix Rousse, Hospices Civils de Lyon, Lyon, France; ^7^Institute of Cognitive Sciences Marc Jeannerod, UMR 5229, CNRS, Lyon, France

**Keywords:** prenatal diagnosis, mother-infant interactions, developmental outcome, perinatal care, mother infant dyad, infant directed speech, langage development

## Abstract

**Introduction:**

Mother–child interactions during the first years of life have a significant impact on the emotional and cognitive development of the child. In this work, we study how a prenatal diagnosis of malformation may affect maternal representations and the quality of these early interactions. To this end, we conducted a longitudinal observational study of mother–child interactions from the gestational stage until the baby completed 12 months of age.

**Participants and Methods:**

We recruited 250 pregnant women from a local university hospital. Among them, 50 mother–infant dyads participated in all stages of the study. The study group consisted of 25 pregnant women with fetuses with some structural alteration and the control group consisted of 25 pregnant women with fetuses without structural anomalies. We collected obstetric and socio demographic data and pregnancy outcomes. Anxiety and depressive state data were collected using the COVI and Raskin Scales. We video-recorded the mother–infant interactions during several stages, including when the child was a newborn and when the child was 2, 4, 6, 9, and 12 months of age. The quality of the mother infant interactions were measured using the Coding Interactive Behavior (CIB). The interactive moments recorded on video was composed of three different activities, each one lasting appoximately 3 min, which included (1) Free Interaction, where the mother was instructed to interact “as usual” without any toy, (2) Toy Interaction, where the mother and baby played with a puppv, and (3) Song Interaction, where the mother and baby interacted while the mother sang the “Happy Birthday” song.

**Results:**

In the gestational phase, there was a significant difference between the groups with respect to anxiety and depression scores, which were significantly higher for the study group. In the postnatal phase, we found significant differences between the groups with respect to CIB scales after the child completed 6 months of age: the study group presented significantly higher values of **Maternal Sensitivity** at 6 months of age, of **Baby Involvement** at 9 and 12 months of age, and of **Dyadic Reciprocity** at 6, 9, and 12 months of age, while the control group presented significantly higher values of **Withdrawal of the Baby** at 6 months of age, and of **Dyadic Negative States** at 6 and 9 months of age.

**Conclusion:**

The support offered by the study favored the mother–infant bond and had a positive effect on the quality of interaction during the first year of life, despite the presence of prenatal diagnosis.

## Introduction

For most women, pregnancy is a period of strong emotions, which undergo changes ranging from positive to negative, often ambivalent. In general, the woman starts having fantasies about how the baby will look, by giving it some characteristics and developing feelings (Klaus and Kennell, 1982).

As the fetus develops, the parents build a network of dreams, secret wishes, memories, and words around it, especially the pregnant woman, who is physically engaged with the fetus (Wilheim, 1997; Bydlowski, 2000). Such processes are called maternal representations. For this reason, these representations commonly alternate and are distinct, complementary, and even antagonistic.

Maternal representations appear long before pregnancy and considerably develop during the transition periods for parenthood (pre, peri, and postpartum), as described by Benedek (1959). The mother can thus represent the fetus and attribute current characteristics to it, projecting their representations on her role and anticipating future interaction. These representations also depend on environmental factors and undergo changes during pregnancy, postpartum and in the parenting process, from the meeting with the real baby, which is different of the imagined baby (Stern, 1991; Ammanitti et al., 1999).

The effect of ultrasound on maternal representations is also relevant, considering the number of pregnant women who claim that after the first ultrasound, they began to really feel pregnant. It is also at this time that they can see that their children are not completely undefined, but already have the form of a human being (Fonseca et al., 2010; Richter et al., 2020).

With the technological advances on prenatal exams, especially the ultrasound with increasingly sharper images, the real presence of the child is increasingly incorporated by the parents and family members. This fact raises questions about the repercussion of these advances on the gestational process and on the stages of attachment, and requires specific studies.

The assessment of mother–child interactions is an important issue in perinatal psychopathology because of its emotional and cognitive impact on child development (American Psychiatric Association, 2000; Viaux-Savelon et al., 2013; Binda et al., 2019; Parlato-Oliveira et al., 2020). Thus, the child will do his/her first acquisitions which are strongly influenced by the gestational period, parental bonding and the family environment. The family is seen as the first social nucleus and the main bond of the child. It plays a fundamental role in the baby’s growth and development, as it provides a stimulating environment for healthy interactions and relationships since pregnancy, especially during the last trimester, which is a stage when it is observed that there are moments of “conversations” between the mother and her fetus (Cohen et al., 2011). This initial language of the mother toward her fetus is important as it structures the representations she has about her future baby.

Viaux-Savelon et al. (2012) in a study with pregnant women, who had minor fetal malformation in their imaging exams, found later that future children were at risk of negative consequences for mother–child early interaction and emotional engagement development. In another study, it was found that in monitored pregnant women, there was an important impact of the diagnosis and prognosis of the malformed fetus, on their family and social relationships, their perceptions about the fetus and the gestational period, as well as on their projections about childbirth and the future (Væver et al., 2020).

The present work aims to investigate the impact of a prenatal diagnosis of malformation on the quality of mother–infant interaction up to the first year of life or 12 months of corrected age in cases of prematurity. Our hypothesis is that such a diagnosis has effects on the quality of mother–infant interaction, but can be influenced by a structured follow-up. To test our hypothesis, we followed the quality of mother–infant interactions in two groups: (1) a study group of mother–infant dyads in a situation of greater emotional fragility due to a prenatal diagnosis of malformation or due to confirmation of a structural alteration, and (2) a control group of dyads whose fetuses did not present structural alteration.

## Materials and Methods

### Study Design, Participants, and Ethics

This longitudinal study began after approval by the Research Ethics Committee (CAAE: 548.79816.0.0000.5149) on March 30, 2016 and was completed in the last quarter of 2019 at the Fetal Medicine Center of the Hospital of the Federal University of Minas Gerais (HC/UFMG). It is a controlled longitudinal study (analytical), a prospective case-control study (concurrent cohort), i.e., an observational study in which the dyad (mother–baby) of the two groups, the study group with fetuses/babies with some structural anomaly and the control group with fetuses/babies without any structural anomalies, were followed longitudinally until the babies reached 12 months of age.

In the first stage of the study, which was the gestational phase between April 2016 and December 2017, we recruited 250 pregnant women at Jenny de Andrade Faria Institute Clinical Hospital/Federal University of Minas Gerais (HC/UFMG). The closing date of the data collection of the first stage followed the closing schedule of the data collection of the second stage, considering that the last monitored babies should complete 12 months of age by December 2018. The pregnant women in both the groups (study and control) were initially selected through data collection from the Jenny de Andrade Faria Institute (HC/UFMG). The researcher participated in a weekly meeting of the fetal medicine team at HC/UFMG in which, in addition to the lectures, all cases monitored by the Center for Fetal Medicine (CEMEFE/UFMG) were discussed.

The necessary ethical measures were taken in accordance with the norms established by the Ethics Committee for Research with Human Beings at UFMG. Potentially eligible pregnant women, along with their partners, if applicable, received verbal and written information about the study. They gave written consent to participate. A specific consent form was signed to authorize the recording of videos of mother–child interactions. Where applicable, the participant’s partner or co-parent signed a consent form for the collection of information about the child and the recording of videos.

Inclusion criteria were: Study Group: Pregnant women enrolled in Prenatal Care at Centro de Medicina Fetal HC/UFMG, who were in the third gestational trimester, i.e., from the 25th gestational week, and who received the prenatal diagnosis of some type of structural anomaly without associated malformations that compromise the neurological, auditory, and visual development; Control Group: Pregnant women enrolled in prenatal care at HC/UFMG, who were in the third gestational trimester, i.e., from the 25 gestational week, with normal imaging tests.

The exclusion criteria for the two groups (study and control) were: babies with severe congenital malformations or genetic syndromes, peri-intraventricular hemorrhage (PHIV) grades III and IV, periventricular leukoencephalomalacia (PVL), severe cardiopathies, cerebral palsy, muscle tone, hearing and/or visual impairment, and Apgar score lower than 7 in the 5th minute. To exclude the possibility of morphological changes in the brain, all children in the study group underwent a transfontanellar ultrasonography (TFUS) examination in the first month of life. Premature children monitored by CEMEFE already undergo routine USTF in the Neonatal Unit. In this case, we always consider the result of the last USTF exam and only those exams performed at 2 weeks of age or more (Leijser et al., 2006, 2007).

### Data Collection Protocol

During their reports, they answered the questions of the Irmag-R (IRMAG: Interview pour les Representations Maternelles pendant la Grossesse (Ammanitti et al., 1999). The IRMAG evaluates maternal representations during pregnancy – how the mother perceives the experience of pregnancy. The survey is composed of 41 questions and is preferably completed during the last trimester of pregnancy. These questions explore seven categories: sophistication of perceptions, openness to change, emotional engagement, coherence, differentiation, social dependence, and sophistication of fantasies. The clinician then evaluates each answer into one of five different degrees: 1-poor, 2-limited, 3-moderate, 4-considerable, and 5-highly accentuated. With this assignment, the clinician can evaluate maternal representations according to a final score, as integrated, moderate, and non-integrated.

We also analyzed IRMAG responses to categorize the maternal representations patterns with two independent scales as recommended by (Ammanitti et al., 1999). Depression Scale: Échelle de Gravité by La Dépression by Raskin and Crook (1976) and Anxiety Scale: Échelle de Gravité by Covi (1986). Next, audio recordings of an interactive moment were made of the mother with the fetus. The postnatal period began at the maternity ward and/or Babylab, and secondary data from the perinatal and postnatal moments were recorded from the analysis of medical records, “Child Health Handbook/Unified Health System – Ministry of Health” (weight, height, head circumference, Apgar score, and complications), and interview with parents (Table 1).

**TABLE 1:** Demographic and clinical information for the mothers and newborns.

	Case (*N* = 25)	Control (*N* = 25)	*p*
**Socio-demographic characteristics**			
Mother’s age (years): mean (SD)	27.7 (6.8)	26.6 (7.2)	0.575
Mother’s years of education: mean (SD)	9.0 (1.8)	9.2 (1.3)	0.288
Mother’s couple status (married/unmarried)	21/4	19/6	0.725
**Pregnancy characteristics**			
Number of pregnancies: mean (SD)	2.6 (1.8)	2.0 (1.3)	0.135
Fetal risk (high/low)	25/0	0/25	<0.001
Maternal risk (high/low)	6/19	5/20	1.000
**Delivery characteristics**			
Type of delivery (vaginal/cesarean)	23/2	25/0	0.490
Gestational age (weeks): mean (SD)	31.3 (5.0)	33.9 (4.8)	0.065
Food at hospital discharge (FBD/BAB)	23/2	25/0	0.490
**Newborn characteristics**			
Gender of the baby (male/female)	13/12	9/16	0.393
Weight (g): mean (SD)	3177.2 (407.1)	3145.4 (404.5)	0.777
APGAR score 1	7.8 (2.0)	8.3 (0.8)	0.930
APGAR score 2	9.0 (0.9)	9.0 (0.6)	0.996
Breastfeeding time (months): mean (SD)	7.7 (3.3)	6.9 (3.0)	0.536
**Psychopathology Covi Raskin**			
Covi (no gravity/serious)	13/12	21/4	0.015
Raskin (no gravity/serious)	15/10	24/1	0.002
Raskin Covi	16/9	24/1	0.005
**CIB – newborn**			
Intrusion: mean (SD)	1.48 (0.77)	1.24 (0.52)	0.204
Affection: mean (SD)	4.04 (0.45)	3.84 (0.62)	0.202
Recognition: mean (SD)	3.88 (0.67)	3.76 (0.72)	0.545
**Maternal representations (Irmag-R Scale: interview for maternal representations during pregnancy)**			
Integrated/balanced (limited/self-oriented/child oriented)	2/6/9	5/5/8	0.529
Reduced/disinvested (accentuated/scared/self-oriented)	1/5/2	1/1/3	
Non-integrated/ambivalent (confuse/absorbed by itself)	0/0	1/1	
Maternal representation of the baby (bad/intermediate/good)	0/8/17	2/5/18	0.123

*Categorical variables: Fisher’s exact test. Mother’s age (normally distributed, Shapiro–Wilk p > 0.05 for study and control groups): Student’s t-test.Other numerical variables (non-normally distributed, Shapiro–Wilk p < 0.05 for at least one group): Wilcoxon test. BFD, breastfeeding on demand. BFS, breastfeeding with supplementation.*

In all meetings with the mother-baby dyad, the baby (newborns, with two, four, six, nine and twelve months of chronological age, or corrected in cases of prematurity) initially underwent an assessment and observation of language development – Protocol adapted for the assessment of children aged 2 to 24 months – Gordo Protocol (Gordo et al., 1994), hearing, through the observation of audiological tests already performed periodically – Otoacoustic emissions - “Universal Neonatal Hearing Screening” and the Observation of Hearing Behavior (Azevedo et al., 1995) and a screening of neuropsychomotor development – Denver Developmental Screening Test II (TTDD II) (Drachler et al., 2007; Leão et al., 2013).

The video of the interactive moment between the mother and the newborn was recorded in the hospital environment before discharge or at the Babylab of the School of Medicine, after hospital discharge. Babylab was a room specially prepared to receive the mother–baby dyad. For example, the room included a baby chair and a mirror in order to ensure the best view of the face and body of both the mother and the baby.

The interactive moment recorded on video was composed of three different activities, each one lasting approximately 3 min, which included (1) **Free Interaction**, where the mother was instructed to interact “as usual” without any toy, (2) **Toy Interaction**, where the mother and baby played with a puppy, and (3) **Song Interaction**, where the mother and baby interacted while the mother sang the “Happy Birthday” song (Ouss et al., 2018).

The videos of the interactive moments were coded/analyzed using the Coding Interactive Behavior (CIB) (Feldman, 1998). CIB is a global classification system for interaction between parents and children and/or between two or more partners, which includes micro-level codes and global rating scales. Each code is rated from 1 (a little) to 5 (a lot). Forty-five different codes have been grouped into various interactive features (Feldman, 1998). In this study, the version for newborns and the version for children aged 2–36 months were used. In order to use CIB, the main researcher of the study received training from Feldman’s team in February 2018 in the city of Lille/France.

The Coding Interactive Behavior – Newborn (CIB for newborns) version is a micro-analytical coding scale (microanalysis) for interacting adults and infants (from 0 to 3 months). This scale was based on the Neonatal Behavioral Assessment Scale (NBAS) – ENAC by Brazelton (1983). It comprises various information from the mother–infant dyad interaction, such as the direction of the mother’s gaze, the mother’s affection, vocalization of the mother, mother’s touch, baby’s position on mother’s lap, baby’s readiness, baby’s affection, baby’s vocalization, and parental scales that are numerical variables (intromission, affection, and recognition).

In the CIB version used from 2 months of age onward, eight domains were analyzed as follows (Viaux-Savelon et al., 2013):

1. **Maternal Sensitivity:** Maternal sensitivity is the average of maternal acknowledgment of the baby’s interactive signals, imitation of the baby’s behavior, appropriate tone of voice, appropriate range of affection, resourcefulness in dealing with negative infant states, and supportive presence (Chronbach’s alpha = 0.965).
2. **Maternal Intrusiveness:** Maternal intrusiveness is the average of maternal inappropriate physical manipulation, mother’s predominant behavior (the degree to which the mother ignores the baby’s signals and interrupts the baby’s ongoing behavior), maternal anxiety, negative maternal affection/anger toward the baby, maternal criticism of infant behavior, and mother-led interaction (the degree to which interactions were judged to be led by the mother’s needs rather than the infant’s needs, pace, and agenda) (Chronbach’s alpha = 0.867).
3. **Maternal Limit Setting:** Maternal limit setting is the average of style consistency, task persistence, and the degree to which the mother persists in a single task.
4. **Baby Involvement:** Baby Involvement is the mean of joint attention, child positive affect, parental affection, alertness, low fatigue, vocalizations/verbal production, initiation, competent use of environment, infant-led interaction, symbolic play, and positive content affect (Chronbach’s alpha = 0.72).
5. **Withdrawal of the Baby:** Withdrawal and baby avoidance express an avoidance toward the mother or a global disengaged behavior from the child, the degree to which the child is not involved, does not participate, and is disconnected from the activity, and the baby’s emotional ability, fatigue, or low level of alertness (Chronbach’s alpha = 0.793).
6. **Baby Compliance et Reliance:** Baby compliance et reliance is the measure of how much the baby collaborates with the mother, depends on the mother, and persists in a single task.
7. **Dyadic Reciprocity:** Dyadic reciprocity is the average of the mother’s elaboration of the baby’s vocalizations and movements, maternal gaze directed at the baby, the child’s gaze directed at the mother or joint activity, verbal praise for the baby’s behavior, affectionate touch and enthusiasm, infant vocalization, warm and positive affect for both parent and child, dyadic adaptation-regulation, and fluency of the interaction (Chronbach’s alpha = 0.965).
8. **Dyadic Negative States:** Dyadic negative states was the mean of negative maternal affect/anger, mother’s hostile behavior, child’s negative emotional affect, dyad constriction, and tension expression (Chronbach’s alpha = 0.793).

In addition to the 45 global codes of the “2 month to 36 month version of the CIB” used in previous studies by Feldman (1998), Keren et al. (2001), and Feldman et al. (2004), We also used the “Newborn version of the CIB”, which was based on the Brazelton Neonatal Behavioral Assessment Scale (Feldman, 1998; Brazelton, 1983), which is 11 items in all, 5 for mothers, such as touch, vocalization, look; 3 for the baby as protitude, vocalization and 3 for the dyad as a unit.

### Statistical Analysis

All analyses were performed at the significance level of 0.05 and implemented using the R programming language. The normality of the distribution for the numerical variables was verified by the Shapiro–Wilk test. Numerical variables with significant evidence of non-gaussianity were expressed in terms of median, first, and third quartiles. The remaining numerical variables were considered normally distributed with their means and standard deviations reported. Categorical variables were represented by their contingency tables.

The hypothesis of different means between the control and study groups was performed for normal and non-normal numerical variables, respectively, using the parametric unpaired and two-tailed Student’s *t*-test and the Wilcoxon–Mann–Whitney non-parametric test. Differences in the contingency tables for categorical variables were evaluated using the Fisher’s exact test.

## Results

**FIGURE 1:**
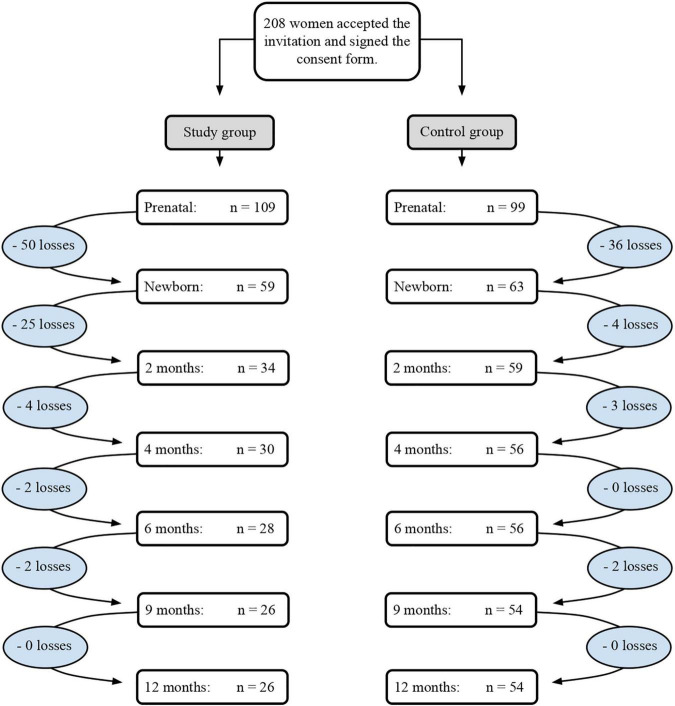
Number of followed dyads until the completion of data collection.

A total of 250 pregnant women from the Jenny Andrade Faria Institute – the prenatal service from the Hospital of the Federal University of Minas Gerais (HC/UFMG) – were invited to participate in the study. Among the 250 women, 208 (109 from the study group and 99 from the control study) agreed to participate, signed the consent form, and completed the first step of the study (Step 1), which involved the audio-recording of the mother–fetus interactive moment. Due to the drop-out of 86 women, 122 dyads (59 from the study group and 63 from the control group) went to the next step of the study (Step 2), which involved the video-recording of the mother–infant interactive moments since the postnatal period. Throughout this step of the study, the number of participants gradually reduced. Figure 1 shows the number of losses in each group and at different stages of the study. We concluded data collection with 80 dyads (26 from the study group and 54 from the control group) and conducted the main study with 25 dyads from each group.

Losses corresponded to different reasons, such as the baby’s or the mother’s death. Exclusion criteria included the presence of severe congenital malformations or genetic syndromes, peri-intraventricular hemorrhage (HPIV) grades III and IV, periventricular leukoencephalomalacia (LPV), severe heart disease, cerebral palsy, altered muscle tone, hearing and/or visual impairment, and an Apgar less than 7 in the 5th minute. In order to exclude the possibility of morphological brain alterations, all children in the study group underwent a transfontanellar ultrasound scan (USTF) in the first month of life.

The dyads of the study group included: (1) eight cases of hydronephrosis (bilateral hydronephrosis with megabladder and urethra, hydronephrosis with mild dysmorphisms, hydronephrosis with gestational toxoplasmosis, and hydronephrosis with heart disease); (2) one case of a single umbilical artery; (3) two pulmonary malformations (suspected cystic fibrosis and lung cysts; pulmonary malformation plus polydactyly); (4) one case of a choroid plexus cyst; (5) one case of myelomeningocele; (6) one case of ventricular dilatation; (7) three cases of increased nuchal translucency (one with polydactyly as well); (8) five cases of isoimmunization; (9) two cases of gestational toxoplasmosis (a mother with Crohn’s disease); and (10) one case of heart disease. All of these cases were assigned to a high degree of risk according to a Brazilian standard (Gomes et al., 2019).

### Pregnant Women Representations During Pregnancy

The final score categorizes women’s representations into three patterns: good (integrated/balanced), intermediate (reduced/disinvested), and poor (non-integrated/ambivalent). Table 1 shows that for the different types of maternal representations there was no statistically significant difference between the study and control groups (*p* > 0.05). However, for all binary variables related to severity scales (with and without severity) there was a significant association (*p* < 0.05) with the groups.

### Maternal Depression and Anxiety During Pregnancy

The association has always been between the class with severity and the study group, pointing to a higher prevalence of severity in the study group when compared to the control for the Covi scores (pregnant women with anxiety severity scores, *p* = 0.015), Raskin scores (pregnant women with depression severity scores, *p* = 0.002) and Raskin associated to Covi scores (pregnant women with scores for severity of anxiety and depression, *p* = 0.005).

### Mother Child Interaction Outcome During the First Year Post Partum

It can also be seen in the newborn stage that for the items observed on the Global Parental Scale (intromission, affection, and recognition) there was no significant difference between the study and control groups. Therefore, regarding the statistical analysis of all variables observed during the moment of free interaction of the mother–newborn dyad, there was no significant difference between the study and control groups.

Through the analysis of the CIB scales – CIB for newborns (mother’s vocalization, mothers’ gaze direction, mother’s affection, mother’s touch and way of holding the baby, baby’s vocalization, baby’s readiness, and baby’s affection) of this moment of interaction of the mother–baby dyad, it was possible to observe that during speech directed to the baby (mother’s type of vocalization) many newborns tried to open their eyes, directed their gaze to the mother’s face and eyes, made a smile, and moved their limbs such as hands and feet. It was also found that many mothers interpreted the baby’s reactions as a response to the content of what was said by her and transmitted to the baby as a dialog and reciprocity. The baby’s reactions, especially the look and smiles, were recognized by the mother as communicative signs, even if subtle and of short duration, as her readiness respects the baby’s sleep/wake cycle, which is characterized at this stage by short waking intervals.

**FIGURE 2:**
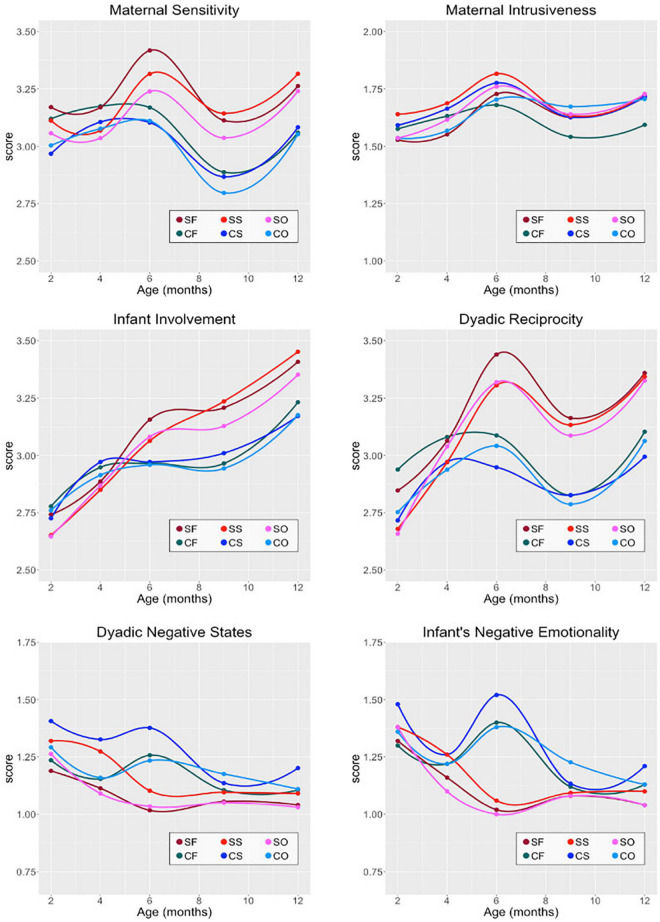
Temporal evolution of Maternal Sensitivity, Maternal Intrusiveness, Infant Involvement, Dyadic Reciprocity, Dyadic Negative States, and Infant’s Negative Emotionality. SF, study group/Free; SS, study group/Song; SO, study group/Object; CF, control group/Free; CS, control group/Song; CO, control group/Object.

Only after 6 months of age was there a statistically significant difference between the groups in the domains: Maternal Sensitivity in the interaction mediated by free maternal speech; Dyadic reciprocity in the situation mediated by free maternal discourse and by the situation mediated by song; Baby’s Negative Emotionality/Baby Withdrawal in situations mediated by free maternal speech, by the object, and by the song; Dyadic Negative States in free and song-mediated situations (Table 2). The highest score in the domains (Maternal Sensitivity, Dyadic Reciprocity) that confer positive characteristics on the quality of the dyad interaction was attributed to the study group. On the other hand, the highest score in the domains (Baby Withdrawal, Negative States of the Dyad) that confer negative characteristics on the quality of the dyad interaction was assigned to the control group. Figure 2 shows in solid circles the values obtained for each score at 2, 4, 6, 9, and 12 months. Even though the measurements are not continuous, smooth curves were fit to the data in order to show the temporal score evolution for the six domains investigated in this study.

**TABLE 2:** Significant differences between study and control groups for the investigated domains.

Domain	Age (month)	Study mean (SD)	Control mean (SD)	Effect size	*p*
Maternal Sensitivity/Free*	6	3.42 (0.35)	3.17 (0.51)	0.57	0.019
Dyadic Reciprocity/Free*	6	3.44 (0.41)	3.09 (0.61)	0.67	0.013
Dyadic Reciprocity/Song	6	3.31 (0.47)	2.95 (0.58)	0.68	0.02
Infant’s Negative Emotionality/Free*	6	1.02 (0.1)	1.4 (0.78)	−0.69	0.01
Infant’s Negative Emotionality/Song*	6	1.06 (0.17)	1.52 (0.76)	−0.84	0.007
Dyadic Negative States/Free*	6	1.02 (0.06)	1.26 (0.48)	−0.7	0.013
Dyadic Negative States/Song*	6	1.1 (0.19)	1.38 (0.48)	−0.75	0.018
Dyadic Reciprocity/Free	9	3.16 (0.52)	2.83 (0.55)	0.63	0.031
Dyadic Reciprocity/Object	9	3.09 (0.52)	2.79 (0.52)	0.57	0.048
Dyadic Reciprocity/Song	9	3.13 (0.53)	2.83 (0.53)	0.57	0.048
Dyadic Negative States/Object*	9	1.05 (0.23)	1.18 (0.41)	−0.38	0.038
Infant Involvement/Free*	9	3.21 (0.33)	2.97 (0.41)	0.65	0.033
Infant Involvement/Song	9	3.24 (0.37)	3.01 (0.39)	0.6	0.039
Dyadic Reciprocity/Song	12	3.34 (0.52)	2.99 (0.6)	0.62	0.034
Infant Involvement/Song	12	3.45 (0.39)	3.17 (0.39)	0.72	0.015

*Non-normally distributed: Wilcoxon test. All other variable normally distributed (t-tests).

Some domains showed a significant difference associated to fetal risk. The Maternal Sensitivity in the free situation (*p* = 0.019) and Dyadic Reciprocity in the free situation (*p* = 0.013) and in the situation mediated by song (*p* = 0.02) were related to higher fetal risk. On the other hand, Baby Withdrawal domains in all situations, free (*p* = 0.01), object (*p* = 0.002), and song (*p* = 0.007), and Dyadic Negative States in the free situation (*p* = 0.013) and in the situation mediated by the song (*p* = 0.018) presented a negative association with fetal risk, i.e., the higher the score for these domains, the lower the fetal risk.

At 9 months of age, some domains, including Involvement of the Baby in the free situation (*p* = 0.026) and in the situation mediated by the song (*p* = 0.039), and Dyadic reciprocity in the free situation (*p* = 0.031) and in the situation mediated by the object (*p* = 0.048) presented significant differences between the groups. In this case, positive characteristics on the quality of the dyadic interaction were observed for the study group on the domains of Dyadic Negative States in the situation of interaction mediated by the object (*p* = 0.038). It was also possible to observe a strong association between domains that confer positive characteristics to interaction, such as Dyadic Reciprocity and Baby Involvement, with an increased fetal risk. However, domains relating negative characteristics to interaction situations, such as Dyadic Negative States, were related to low fetal risk and associated to the mother’s marital status (at 6 months: *p* = 0.006, and at 9 months: *p* = 0.009; *p* = 0.006), specifically when the mother was single and the father of the baby was not present.

At 12 months of age, we observed that domains related to the Involvement of the Baby in the situation mediated by the song (*p* = 0.015) and Dyadic Reciprocity in the situation mediated by the song (*p* = 0.034) presented higher scores for the study group, meaning a quality interaction. These domains were also significantly associated with increased fetal risk. On the other hand, the Dyadic Negative States domain mediated by the song presented higher scores for the controls, with a significant association with lower fetal risk (*p* = 0.024).

Regarding demographic data and pre, peri, and postnatal variables, there was a significant difference between the groups in the variable “fetal risk” (*p* = 0.001) and for the anxiety (Covi scale, *p* = 0.015) and depression (Raskin scale, *p* = 0.002) score.

From the three different interaction situations, we observed that the situation of free interaction and the one mediated by the song were more present in the domains that showed a significant difference between the groups.

## Discussion

The purpose of this work was to study if the prenatal diagnosis of malformation could affect the mother representations and the quality of the early interactions between the mother and her child. To this end, we recruited 50 mothers, of which 25 received such a diagnosis (the study group) and 25 did not (the control group), and we measured the quality of mother–baby interactions using the CIB (Feldman, 1998) at six stages: when the child was a newborn and when the child was 2, 4, 6, 9, and 12 months of age. Additionally, before the birth of the child, we measured the degree of depression and anxiety of the mother using the Raskin and Crook (1976) and Covi (1986) scales. In the following paragraphs we discuss the obtained results.

During the gestational phase, the scores of anxiety and depression were significantly higher for the study group. This result is in line with the literature. Cunha et al. (2016) and Kaasen et al. (2017) show a strong correlation between the prenatal diagnosis of malformation and levels of anxiety and depression of the mother. To asses these levels, Kaasen et al. (2017) used forms of self-report [Impact of Event-Scale-22 (IES) and Edinburgh Postnatal Depression Scale], while Cunha et al. (2016) used the Beck scales (Beck Anxiety Inventory and Beck Depression Inventory).

When the baby was a newborn, we could notice from our CIB analysis that many mothers interpreted the reactions of the baby as a reply to what was spoken to the baby. Thus, the mothers interpreted the interaction as a form of dialog (Brazelton and Cramer, 1992). This observation is supported by the literature, where Trevarthen and Delafield-Butt (2016) state that the newborn is intuitively social, searching for affective relations with partners willing to share the pleasure of making and discovering with “human sense.”

When the baby was 2 months of age, we could not find any statistically significant difference between the study and control groups. However, we observed that, at this age, some babies were already actively responding to the mother’s interactive exchange initiatives, mainly by directing and continuing to look at the mother’s face and eyes, smiles, and vocalizations, while other babies systematically avoided the exchange of glances, even keeping their heads turned to the side as a gesture of refusal, escape, or withdrawal. In these situations, the mother insisted and even centered her head, raising the baby in front of her eyes, but even so, the child kept looking away or keeping the head turned to one side. Colonnesi et al. (2012) state that the direction of the eyes and the performance of expressive movements and vocalizations are the first ways in which the baby communicates emotionally. Bradshaw et al. (2021) show that differences in social communication are a defining and robust characteristic of autism spectrum disorder (ASD). More specifically, Bradshaw et al. (2021) show that typical babies usually combine their vocalizations with a gaze toward the parents, while babies that were ulterior diagnosed with ASD were less prone to do so.

It was only after 6 months of age that we found statistically significant differences between the study and control groups with respect to the CIB scales. The study group presented significantly higher values of **Maternal Sensitivity** at 6 months of age, of **Baby Involvement** at 9 and 12 months of age, and of **Dyadic Reciprocity** at 6, 9, and 12 months of age. The control group presented significantly higher values of **Withdrawal of the Baby** at 6 months of age, and of **Dyadic Negative States** at 6 and 9 months of age. Therefore, from 6 months on, mother–baby interactions in the study group were more positive than the mother–baby interactions in the control group. To the best of our knowledge, this is a novel result. One explanation for this phenomenon comes from Brazelton (1988), where he explains that the depression of the mother has a positive side because her hypersensibility urges a search for ways to understand the baby.

In addition to Brazelton’s explanation, we believe that the generally more positive mother–baby interactions from the study group can be understood when we look at the evolution of the mother’s attention to the baby. When the child is born, the mother is endowed with a naturally intense feeling of care toward the baby. During the upcoming months, as the baby becomes less dependent, the mother will gradually de-escalate her attention. We believe that mothers from the study group preserve that intense attention from the early months for a longer period than the mothers from the control group. This hypothesis goes in line with the fact that we observe statistical differences between the groups only after 6 months of age. Indeed, it is at this stage that the baby increases its repertoire of vocalizations and becomes capable of sitting, grabbing objects, and moving with agility (Rothbart and Bates, 2006; Gopnik, 2009; Klein et al., 2009; Trevarthen et al., 2019). The follow-up of this study provided parents with a support structure for their interactions with the baby. Therefore, the more intense the mother–infant interaction, the more the baby will benefit from these new and richer forms of expression.

At 9 and 12 months of age, the significant difference between the groups for the **Dyadic Reciprocity** and **Baby Involvement** domains revealed the presence of characteristics of these stages of development that favored an increase in the frequency of some items, such as “joint attention,” “kid’s relation to the father,” “competence to explore the environment,” and “initiative of the baby,” which give greater quality to the interaction. We believe that the context of care and attention may have favored the interactive domains/constructions of the baby and the dyad. The focus for this developmental issue may have potentiated the baby’s and the dyad’s interactive domains.

The support of the health team, offering an opportunity to embrace and overcome, in the different stages of development (pre, peri, and postnatal stages), may have been decisive and essential for the results of the present study.

According to Shonkoff (2011, 2017), typically developing children are usually taken to health professionals when they become ill or to comply with the early childhood vaccination schedule. In this case, the context of health and development has a more superficial connotation and attitudes of waiting in the face of some intercurrence. Children who need some follow-up in specialized health centers have a life story that involves positive and negative experiences that they will have to overcome and/or celebrate (Shonkoff, 2011, 2017).

Health professionals (Fonseca et al., 2010; Shonkoff, 2011, 2017; Shonkoff and Bales, 2011) know the importance of risk indicators and the need and importance of follow-ups. This knowledge is transmitted to the family members who, in turn, must be well-oriented and concerned with the importance of maintaining consultations, exams, and interventions.

Thus, the difference found between the study and control groups highlights the importance of monitoring and individualized care with the mother–baby dyad from prenatal care and the recognition of the baby as an active participant in the dyad.

### Study Limitations

We identify at least two study limitations: (1) the number of participants who completed all the phases of the study, 50 in total, and (2) the diversity of malformation diagnoses, which makes the study group non-homogenous. Even though the number of participants was relatively small when compared to other longitudinal studies, we believe that it is already a remarkable achievement to have concluded with 50 dyads. Given the duration of this study, a great level of motivation was demanded from the part of the mothers. Their steady presence at several encounters throughout the 12 months was possible only due to the development of a strong relation between the mother and the clinician.

## Conclusion

The longitudinal study from the gestational phase to the baby’s 12 months, with the use of different analysis instruments, allowed to find significant differences between the research group and the control group. It shows the relevance of reception and care for development, especially for the most vulnerable populations.

We conclude that “the effects of prenatal diagnosis” became evident at some stages of development, and follow-up may have favored the significant differences that improved the quality of mother–infant dyad interactions. This result is an essential finding for understanding the communicative resources used by the baby and its mother in the complexity of the synchrony of the first relationships.

Therefore, the present study recognizes the need for more studies, with a larger sample, increasingly enabling a better understanding of this initial phase of the baby’s development in the interactive relationship with the mother.

## Data Availability Statement

The raw data supporting the conclusions of this article will be made available by the authors, without undue reservation.

## Ethics Statement

The studies involving human participants were reviewed and approved by the Ethics Committee CAAE: 548.79816.0.0000.5149. Written informed consent to participate in this study was provided by the participants’ legal guardian/next of kin.

## Author Contributions

VS, EP-O, LA, and SVS contributed to the conception and design of the study. VS organized the database and wrote the first draft of the manuscript. AM performed the statistical analysis. VS, EP-O, SVS, and AM wrote sections of the manuscript. All authors contributed to the review of the manuscript, read, and approved the submitted version.

## Conflict of Interest

The authors declare that the research was conducted in the absence of any commercial or financial relationships that could be construed as a potential conflict of interest.

## Publisher’s Note

All claims expressed in this article are solely those of the authors and do not necessarily represent those of their affiliated organizations, or those of the publisher, the editors and the reviewers. Any product that may be evaluated in this article, or claim that may be made by its manufacturer, is not guaranteed or endorsed by the publisher.
